# Selective Occupation by E2F and RB of Loci Expressed by RNA Polymerase III

**DOI:** 10.3390/cancers16030481

**Published:** 2024-01-23

**Authors:** Rebecca E. Sizer, Sienna P. Butterfield, Lucy A. Hancocks, Leonor Gato De Sousa, Robert J. White

**Affiliations:** Department of Biology, University of York, York YO10 5DD, UK; rs1613@york.ac.uk (R.E.S.);

**Keywords:** E2F, p130, RB1, RMRP, RN7SL, RNA polymerase III, tRNA

## Abstract

**Simple Summary:**

RNA polymerase (pol) III synthesizes essential and abundant non-coding RNAs, including all tRNAs. This activity can be constrained by RB, an action that may help to limit cell growth and proliferation. Previous work established that RB binds and represses TFIIIB, a factor required for pol III to transcribe any of its target genes; this explains the apparently universal effect of RB on the pol III transcriptome. We find E2F bound to chromatin in close proximity to many pol III-transcribed genes, where it may recruit RB to influence local chromatin and control the production of specific products. RB recruitment to subsets of pol III-dependent genes provides the potential for more selective regulation than the general dampening of output that can be achieved through interaction with TFIIIB. The regulatory impact of E2F may therefore be strengthened by differentially influencing levels of key non-coding RNAs such as individual tRNAs.

**Abstract:**

In all cases tested, TFIIIB is responsible for recruiting pol III to its genetic templates. In mammalian cells, RB binds TFIIIB and prevents its interactions with both promoter DNA and pol III, thereby suppressing transcription. As TFIIIB is not recruited to its target genes when bound by RB, the mechanism predicts that pol III-dependent templates will not be occupied by RB; this contrasts with the situation at most genes controlled by RB, where it can be tethered by promoter-bound sequence-specific DNA-binding factors such as E2F. Contrary to this prediction, however, ChIP-seq data reveal the presence of RB in multiple cell types and the related protein p130 at many loci that rely on pol III for their expression, including RMRP, RN7SL, and a variety of tRNA genes. The sets of genes targeted varies according to cell type and growth state. In such cases, recruitment of RB and p130 can be explained by binding of E2F1, E2F4 and/or E2F5. Genes transcribed by pol III had not previously been identified as common targets of E2F family members. The data provide evidence that E2F may allow for the selective regulation of specific non-coding RNAs by RB, in addition to its influence on overall pol III output through its interaction with TFIIIB.

## 1. Introduction

Retinoblastoma is a pediatric tumor of the retina that is caused by loss of the *Rb1* gene [1]. Inactivating mutations in this gene also occur in many other tumor types, such as bladder carcinomas and small-cell lung cancers [2,3]. Whereas homozygous deletion causes mouse embryos to die during gestation, heterozygous mice survive but are strongly predisposed to cancer [4,5,6,7,8]. Introduction of exogenous *Rb1* into tumor cells can inhibit growth, proliferation, anchorage-independent colony formation and tumorigenicity in mice [9,10,11]. Such observations demonstrate the potency of this tumor suppressor.

The best-characterized binding partner of the *Rb1* gene product, RB, is the E2F transcription factor, which consists of obligate heterodimers between members of the E2F and DP families [12,13,14]. RB masks the transactivation domain of E2F and recruits chromatin-modifying proteins that can suppress transcription, including histone deacetylases, DNA methyltransferases and the histone methyltransferase SUV39H1 [15,16,17,18,19,20,21,22,23,24,25,26,27,28,29]. Many of the genes that are bound by E2F and repressed by RB encode products that promote nucleotide synthesis, DNA replication and cell cycle progression [30]; a key example is the cyclin E gene, which is overexpressed in fibroblasts from *Rb1*-knockout mice, relative to wild-type [31,32]. Repression of such genes is believed to be pivotal for the ability of RB to inhibit cell proliferation [2]. Conformational changes occur in RB when it is phosphorylated by cyclin-dependent kinases (CDKs), resulting in its release from E2F and allowing cell cycle progression [2,33,34].

RB has been shown to bind a great many partners in addition to E2F [35,36]; indeed, interaction databases list more than 300 human proteins that associate with RB [37]. Amongst these partners is the transcription factor TFIIIB, which is composed of three essential subunits—Brf1, Bdp1 and the TATA-binding protein TBP [38]. Each of these subunits has been detected in complex(es) with RB [39,40,41,42,43,44,45,46]. These interactions were identified using recombinant polypeptides and also with endogenous proteins from mouse and human cells. TFIIIB is required to recruit RNA polymerase (pol) III to all of its template genes, allowing for the synthesis of an eclectic mix of short non-coding RNAs, the most abundant of which are tRNA, 5S rRNA and 7SL RNA [47]. Expression levels of pol III products can influence cell growth and proliferation, as well as oncogenesis [48,49,50,51,52,53,54]. Functional assays demonstrated that RB compromises the ability of TFIIIB to support transcription [39,40,55]; this can explain the inhibitory effect of RB, both in vitro and in vivo, on the transcription of all pol III-dependent genes tested, as TFIIIB is essential in every case [29,39,40,41,42,43,45,46,55,56,57,58]. Nuclear run-on assays revealed that endogenous RB suppresses the synthesis of tRNA and 5S rRNA in mouse embryonic fibroblasts (MEFs), especially in the quiescent state [43,56]. Serum withdrawal causes a ~2-fold decrease in tRNA synthesis in *Rb^+/+^* MEFs, but this response is lost in MEFs from Rb^−/−^ mice, showing that RB is required for the repression of pol III transcription that accompanies quiescence [43]. TFIIIB binds selectively in fibroblasts to hypophosphorylated forms of RB, which are maximal in G0 and early G1 phases [43]. When serum-starved fibroblasts are stimulated to resume proliferating, TFIIIB dissociates from RB at the G1/S phase transition as RB undergoes phosphorylation by CDKs, and transcription by pol III increases in parallel [43]. That RB phosphorylation is directly responsible for this dissociation was confirmed in vitro, where binding to TFIIIB can be blocked if recombinant RB is pretreated with recombinant CDK4 in complex with cyclin D1 and CDK2 in complexes with cyclins A and E [43]. In transfected fibroblasts, expression of VA1, a pol III-dependent adenoviral gene, can be strongly stimulated by co-transfection of CDK2 and CDK4 with cyclins D1 and E; this response can be prevented by the CDK inhibitor p16 [43]. Phosphorylation of RB can be induced in mouse hearts by transverse aortic constriction, and this results in release of RB from TFIIIB and increased tRNA synthesis, effects that are not seen in mice that are null for cyclin D2 [46]. These data support a model in which RB represses transcription by pol III during G0 and early G1 phases through interaction with TFIIIB and then dissociates at the G1/S transition as a result of phosphorylation by CDK4 and CDK2, allowing for the increased production of pol III products.

The functions of RB overlap substantially with those of p107 and p130, with which it shares the bipartite protein-binding domain referred to as the pocket [35,59]. All three “pocket proteins” can inhibit cell growth and proliferation when overexpressed in cancer cells, an effect involving G1-specific cell cycle arrest [11,60,61,62]. They all can also bind to TFIIIB and repress pol III transcription in vitro and in vivo [42,43]. Redundancy is strong between p107 and p130 proteins, which are ~50% identical to each other, but only 30–35% identical to RB. In consequence, mice lacking either p107 or p130 develop normally, although the absence of both results in death soon after birth [63]. In contrast, p107 and p130 cannot compensate for loss of RB during early development, and RB null mice die during midgestation, with defects in the proliferation and/or differentiation of certain cell lineages [4,5,64]. In murine fibroblasts, loss of RB has a greater effect on pol III output than that of p107 and p130 [42]. The tumor suppression function(s) of RB are also considered non-redundant based on its frequent mutation in human cancer, whereas mutations affecting p107 or p130 are rare [65].

It has been suggested that the repression of pol III-mediated transcription may contribute to the ability of RB to suppress cell growth and carcinogenesis by limiting production of key non-coding RNAs, such as tRNAs [48,66,67]. Indeed, growth of cells in culture and in fly embryos, colonies on soft agar and tumors in mice can be stimulated by raising expression of individual tRNAs [68,69,70,71,72,73,74,75,76,77]. Furthermore, cancer survival of human populations correlates with levels of particular tRNAs [52,76,78,79,80]. Other pol III products have also been implicated in cancer progression, such as 7SL (RN7SL) RNAs [81] and RMRP RNA [82,83,84,85,86]. The potency of RB as a tumor suppressor must be strengthened by its ability to inhibit the pol III-dependent synthesis of ncRNAs such as these, which are potentially oncogenic. The restraining effect of RB on pol III output is abolished by point mutations that arose in cancers and by viral oncoproteins that target its pocket domain [41,42,44,56,57,58].

**Figure 1:**
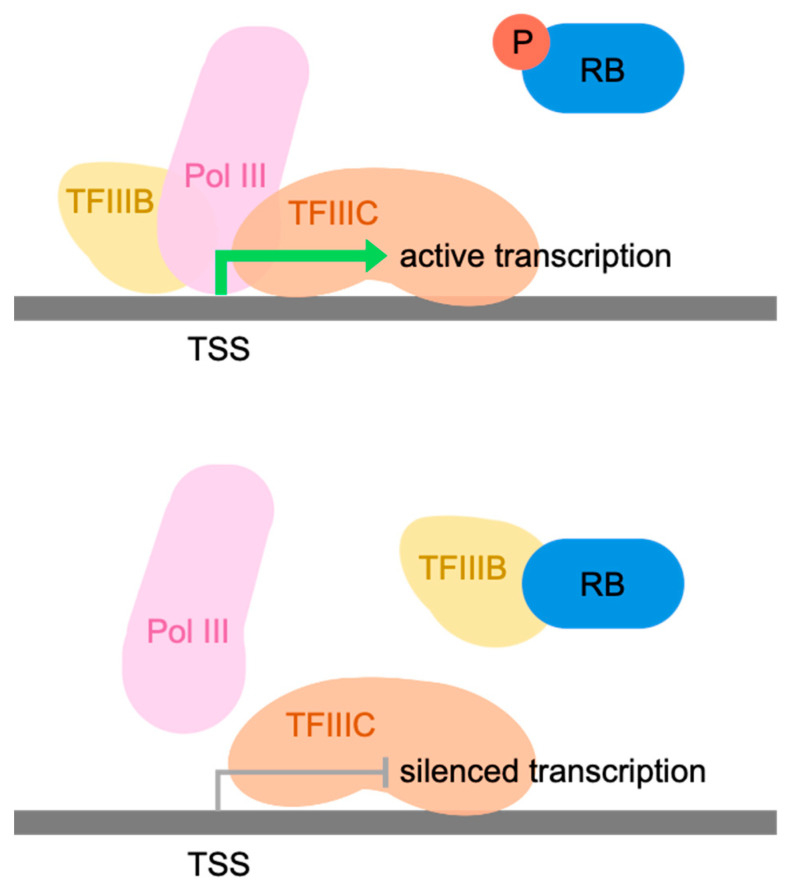
Schematic representation of the original model of Pol III repression by RB. When dephosphorylated, RB associates with TFIIIB, blocking its interactions with TFIIIC and Pol III; prevention of these interactions precludes formation of the preinitiation complex. In this way, RB can inhibit transcription (**bottom**), an effect not observed when RB is phosphorylated (**top**).

Promoters of most pol III-transcribed genes are recognized by the DNA-binding factor TFIIIC, which then recruits TFIIIB through protein/protein interactions [87,88,89]. TFIIIB is necessary and sufficient to recruit pol III to any of its templates, positioning it at the transcription start site [90]. The binding of RB to TFIIIB disrupts its interactions with TFIIIC and pol III, both in vitro and in vivo [91]. These effects are very specific as they are absent in RB mutants with single residue substitutions (567L and 706F) that arose in cancers [91]. On the basis of these data, a model was proposed in which RB disrupts two key steps in assembly of the preinitiation complex—recruitment of TFIIIB by TFIIIC and recruitment of pol III by TFIIIB (Figure 1). A notable feature of this model is that RB is not retained at the pol III-transcribed genes that it represses. This is different from its interaction with genes that are regulated by E2F, which remains bound to promoter DNA whilst also binding RB, thereby providing opportunity for the latter to recruit chromatin-modifying co-repressors [15,16,17,18,19,20,21,22,23,25,26,28].

Repression of the U6 snRNA genes was found to deviate from the general mechanism depicted in Figure 1 [45,55]. This reflects the distinct promoter organization and transcription factor requirements of U6 genes [92]. Whereas the large majority of pol III templates have promoters within their transcribed regions that recruit TFIIIC and then a TFIIIB complex comprising Brf1, TBP and Bdp1, U6 promoters lie upstream of the start site and are recognized by SNAPc, which assists recruitment of a distinct TFIIIB complex in which Brf1 is replaced by the related factor Brf2 [38,93]. Hirsch et al. demonstrated that although RB binds Brf1, TBP and Bdp1, it does not bind Brf2 [45]. However, SNAPc interacts with RB and can recruit it to a U6 promoter [45,55]. They detected no RB at VA1 or tRNA^Lys^ promoters, consistent with the original model, but RB was detected clearly and specifically at a U6 promoter [45]. It was shown to recruit DNA methyltransferases and induce methylation of a specific CpG in the U6 promoter, although this site is not required for RB-mediated repression [29]. Sequential ChIP revealed that RB can co-occupy a U6 promoter with pol III and repress it [45]. The authors concluded that U6 repression by RB utilizes a mechanism that is distinct from that characterized at VA1 and tRNA genes, likely reflecting the use of different basal factors.

The presence of RB at some U6 promoters was subsequently observed in ChIP-seq data from IMR90 fibroblasts [92]. This analysis also detected RB at a few other pol III-transcribed genes with upstream promoters like U6 that recruit SNAPc and Brf2 [93,94], such as the 7SK and RMRP genes. Although this was predictable, given the shared promoter arrangement and factor requirements of this group, the study discovered the additional presence of RB at a subset of tRNA genes (tDNAs), demonstrating further diversity in its interaction with pol III-transcribed genes [92].

We have analyzed ChIP-seq datasets from previous investigations and confirmed the presence of RB at many tDNAs in human IMR90 and BJ fibroblasts, K562 hematopoietic cells and RPE1 retinal cells, amongst others. The presence of tDNAs of the RB-related protein p130 is also observed. An explanation is suggested by the discovery of E2F proteins at sites occupied by RB and p130 in proximity to many tDNAs. Phosphoresistant mutations in RB that enhance its interaction with E2F also increase its occupancy of many tDNA loci. In light of these findings, the original model of pol III regulation by RB should be amended to include its tethering at some tDNA loci by DNA-bound E2F. This opens the possibility of RB-dependent epigenetic regulation, as described for many established E2F targets.

## 2. Materials and Methods

### 2.1. ChIP-Seq Data

#### 2.1.1. Downloading ChIP-Seq Datasets

RB-G (growing), RB-Q (quiescent), RB-S (senescent), p130, p107, FLAG-RB-WT, FLAG-RB-Δcdk and E2F ChIP-seq datasets were obtained from the National Center for Biotechnology Information (NCBI) Sequence Read Archive (SRA) Run Selector (accession: PRJNA734617, accession: PRJNA122045, accession PRJNA147251). Sources are listed in Table 1. Reads were extracted in FASTAQ format using the SRA toolkit in galaxy (https://github.com/ncbi/sra-tools accessed 1 February 2023) [95]. Bowtie2 was used to map reads against the human reference genome and convert the file into a BAM format [96,97]. RB1, control IP, E2F1, E2F4, and E2F5 ChIP-seq files from K562 cells were downloaded in BAM format from the ENCODE portal [98]. The hg19 and hg38 tDNA sequences (*n* = 606 or *n* = 636, respectively), and sno-miRNA sequences (*n* = 2272 and *n* = 2320, respectively) were acquired from the UCSC Table Browser [99].

#### 2.1.2. Quantification of ChIP-Seq Signals Using Easeq

Quantification of ChIP-Seq reads was completed in interactive ChIP analysis software EaSeq (v. 1.111) [100]. BAM files containing ChIP-seq filtered alignments were loaded as “Datasets” into Easeq (available at https://easeq.net accessed 1 February 2023). tRNA genes and sno-miRNA genes were imported as “Regionsets”. ChIP-seq peaks at tRNA genes ± 500 bp from the center were quantified using the “Quantify” tool. Counts were normalized to DNA fragments and reads were normalized to reads per million (within EaSeq’s quantification tool).

#### 2.1.3. Data Analysis

##### Heat Maps

All heatmaps were generated using the “heatmap” function provided by EAseq. tRNA genes or sno-miRNA genes were aligned at their center point, and the signal intensity of transcription factor binding was plotted across 10,000 bp either side. Signal intensity was segmented into 200 bins and sorted according to increasing intensity, calculated using the “Quantify” function provided in EAseq.

##### Line Tracks 

The “Average” function on EAseq was used to visualize signal intensity of binding at all tRNA genes or sno-miRNA genes ± 10,000 bp. The “LineTrack” function on EAseq was used to visualize signal intensity of binding at specific pol III transcribed genes of interest. These genes were gated using the “gate” function, and 10,000–20,000 bp either side were plotted for both strands. Signal intensity was segmented into 400 bins and smoothed for 1 bin.

##### Binding Overlap Analysis 

Quantified values (Q values) were exported to Excel (v. 1808), and tRNA genes with a value above the threshold (average binding) were determined to be bound by that binding factor. Overlap in binding was calculated in Excel. 

**Table 1:** Sources and accession numbers of ChIP-seq datasets.

Source	Cell Type	ChIP-Seq File Type	Accession
Sanidas et al., 2022 [101]	BJ cells	FLAG-RB-WT	SRR14713166
		FLAG-RB-Δcdk	SRR14713167
	RPE cells	FLAG-RB-WT Input	SRR14713168
		FLAG-RB-Δcdk Input	SRR14713169
		FLAG-RB-WT	SRR14713063
		FLAG-RB-Δcdk	SRR14713064
		FLAG-RB-WT Input	SRR14713079
		FLAG-RB-Δcdk Input	SRR14713080
		E2F1	SRR14713081
Michael Snyder, Stanford. 2017	K562 cells	RB1	ENCFF305NFS
Richard Myers, HAIB. 2023		E2F1	ENCFF183WQN
			ENCFF193ODF
		E2F4	ENCFF706ZTX
			ENCFF749XCO
		E2F5	ENCFF027ECD
			ENCFF915WXK
Chicas et al., 2010 [102]	Growing IMR90 cells	RB	SRR034478
			SRR034479
		Mock	SRR034492
	Quiescent IMR90 cells	RB	SRR034480
			SRR034482
		Mock	SRR034493
	Senescent IMR90 cells	RB	SRR034484
			SRR034486
		Mock	SRR034494
	Quiescent IMR90 cells	p130	SRR034483
Ferrari et al., 2012 [103]	Quiescent IMR90 cells	p107	SRR350272

### 2.2. ChIP-Atlas Data

Peak call data for RB, p107 (RBL1), p130 (RBL2), E2F1, E2F4 and E2F5 were obtained from the ChIP-Atlas database (https://chip-atlas.org/ accessed 9 September 2023) using the “Peak Browser” function. The ChIP-Atlas database integrates almost all publicly available ChIP-seq datasets and subjects it to peak calling with MACS2, allowing for visualization of genome-wide binding data for transcriptional regulators. Peak call data in BED format (Q value < 1 × 10^−5^) were used in this study and displayed in the genome browser IGV at regions of interest. Data were aligned to human genome hg38.

## 3. Results

### 3.1. RB and p130 Associate with Many tRNA Genes in Human Fibroblasts

**Figure 2:**
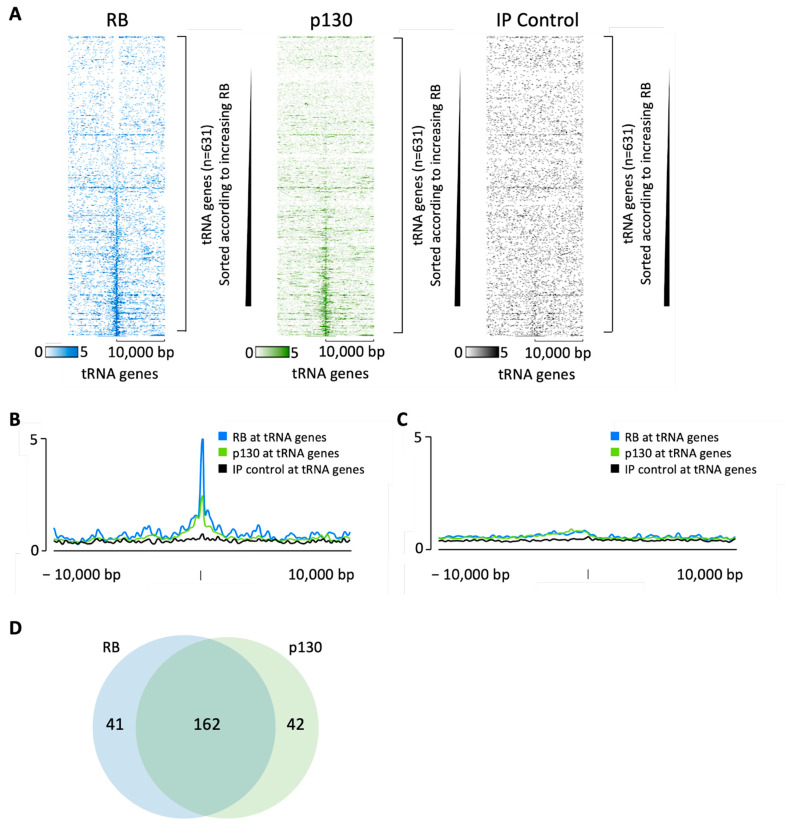
RB and p130 bind to tRNA genes in serum-deprived quiescent IMR90 cells. (**A**) Heatmaps depicting RB (blue), p130 (green) and no-antibody control (black) binding 10 kb either side of hg38 tRNA genes in IMR90 cells. In each heatmap, the tDNAs are sorted (top to bottom) in order of increasing RB signal. (**B**,**C**) Average signal intensity of RB (blue), p130 (green) or mock (black) binding at tRNA genes (**B**) or sno-miRNA genes (**C**) in IMR90 cells. (**D**) Overlap in RB and p130 binding at tRNA genes.

Chicas et al. [102] conducted ChIP-seq analyses of endogenous RB and p130 in IMR90 human diploid fibroblasts. When these data were searched for tDNA loci, heatmaps revealed a concentration of both pocket proteins at a subset of tRNA genes, relative to 10 kb of flanking DNA upstream and downstream (Figure 2A). In each heatmap, the tDNAs are arranged in order of increasing RB signal (top to bottom) and it is apparent that most cases of p130 occupancy overlap with that of RB. Average signal intensity plots confirm the selective localization of RB and p130 at tDNAs, relative to the surrounding regions (Figure 2B). Specificity is demonstrated by the minimal association with miRNA and snoRNA genes (Figure 2C). Approximately 80% of the tDNAs that recruit RB also recruit p130, but these pocket proteins were also each found individually at subsets of tDNAs where the binding of the other did not reach the threshold (Figure 2D). The third pocket protein, p107, can also be detected at a subset of tDNAs in IMR90 cells (Supplementary Figure S1).

**Figure 3:**
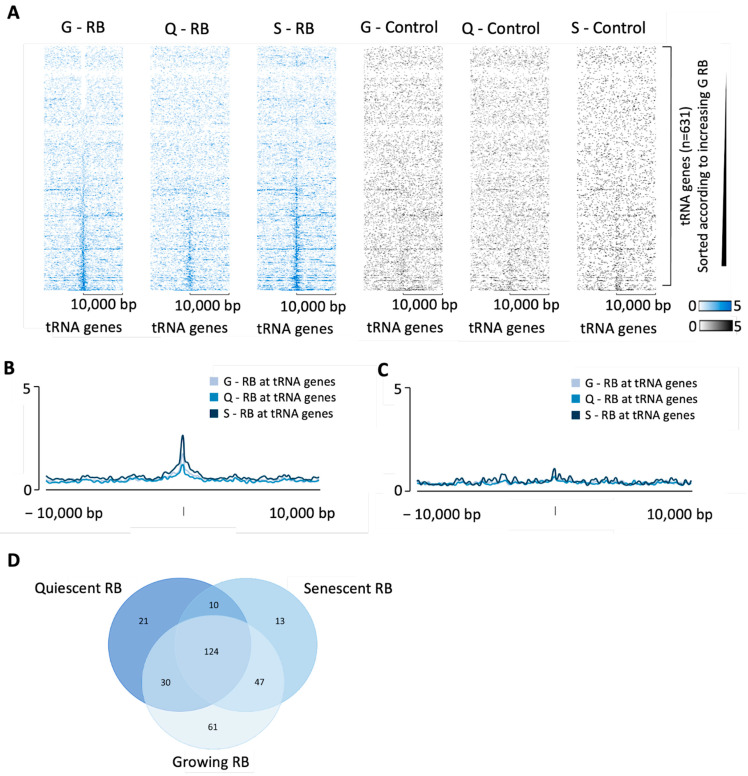
RB binding in growing, quiescent or senescent IMR90 cells. (**A**) Heatmaps depicting RB (blue) or no-antibody control (black) binding 10 kb either side of hg38 tRNA genes in growing (“G”), quiescent (“Q”) or senescent (“S”) IMR90 cells. In each heatmap, the tDNAs are sorted (top to bottom) in order of increasing RB signal in the growing cells. (**B**,**C**) Average signal intensity of RB (**B**) or control (**C**) enrichment at tRNA genes in growing, quiescent or senescent IMR90 cells. (**D**) Overlap in RB binding at tRNA genes in growing, quiescent, or senescent IMR90 cells.

The above data were obtained from IMR90 cells that had been made quiescent by serum deprivation. Chicas et al. also examined RB occupancy when these cells were actively proliferating or undergoing oncogene-induced senescence [102]. Endogenous RB is associated with many tDNAs under each of these conditions, with the strongest enrichment in senescent cells (Figure 3A,B). In contrast, RB is associated with only a small minority of miRNA and snoRNA genes under any of these conditions (Figure 3C). Although the sets of tDNAs bound by RB in proliferating, quiescent and senescent cells overlap substantially, many examples were also detected in which occupancy was strongest for one or two of the conditions examined (Figure 3D). Comparison of the heatmaps (Figure 3A), which were all sorted by strength of RB binding in growing cells, suggests that binding differences between the cell states are primarily due to quantitative relative changes in occupancy strength, rather than substantial qualitative variations between the sets of target genes.

### 3.2. Phosphoresistant Mutation Stimulates Binding of RB to tRNA Genes

**Figure 4:**
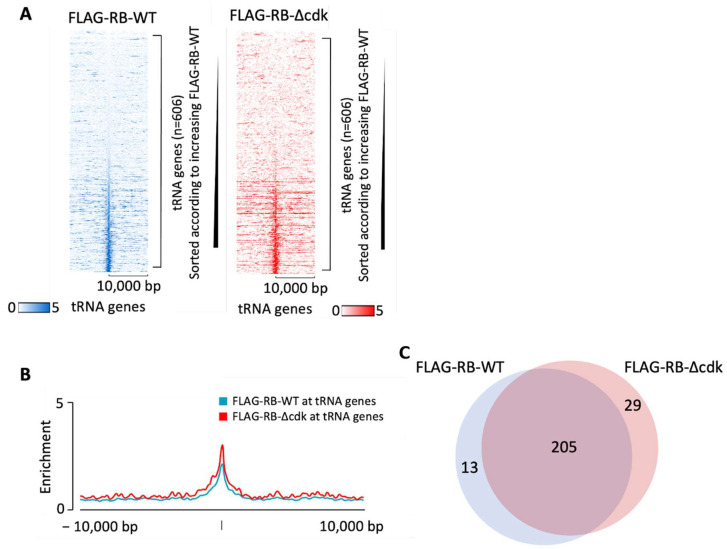
RB binds to tRNA genes in BJ cells. (**A**) Heatmaps depicting binding of FLAG-RB-WT (blue) or FLAG-RB-Δcdk (red) 10 kb either side of hg19 tRNA genes in BJ cells. (**B**) Average signal intensity of FLAG-RB-WT binding (blue) or FLAG-RB-Δcdk (red) binding at tRNA genes in BJ cells. (**C**) Overlap in tRNA genes bound above the threshold by FLAG-RB-WT and FLAG-RB-Δcdk in BJ cells.

To test if RB recruitment to tDNAs is an idiosyncrasy of IMR90 cells, we utilized an orthogonal dataset from an independent study with BJ fibroblasts [101]. In this case, the endogenous RB was replaced by a FLAG-tagged version expressed at comparable levels to improve signal relative to background [101]. ChIP using anti-FLAG antibody revealed association of the tagged RB at many tRNA genes (Figure 4A). This strengthens confidence in the results in Figure 2 and Figure 3. The data provide evidence that RB interacts with multiple tDNAs in two unrelated lines of human diploid fibroblasts. Most tRNA genes that associate with RB above the threshold are common between BJ and IMR90 fibroblasts (Supplementary Figure S2).

Phosphorylation by cyclin-dependent kinases (CDKs) has been shown to regulate the conformation, interactions and activity of RB [2,33,37]. Sanidas et al. [37] created a mutant form of RB, termed RB∆cdk, in which all fourteen known CDK phosphoacceptor sites were substituted with alanine residues that cannot be phosphorylated; they then replaced endogenous RB in BJ cells with a FLAG-tagged version of this phospho-resistant mutant, expressed at similar levels. We analyzed their ChIP-seq data [101] to determine if ablating CDK-mediated phosphorylation affects the recruitment of RB to tRNA genes. Indeed, binding was enhanced by the mutations (Figure 4A,B). As well as causing stronger association in the majority of cases, the mutations also altered a few cases of tDNA selection so that recruitment to subsets of tDNAs was either increased or decreased (Figure 4C). However, most tDNAs bound by wild-type RB were also bound by RB∆cdk and vice versa.

**Figure 5:**
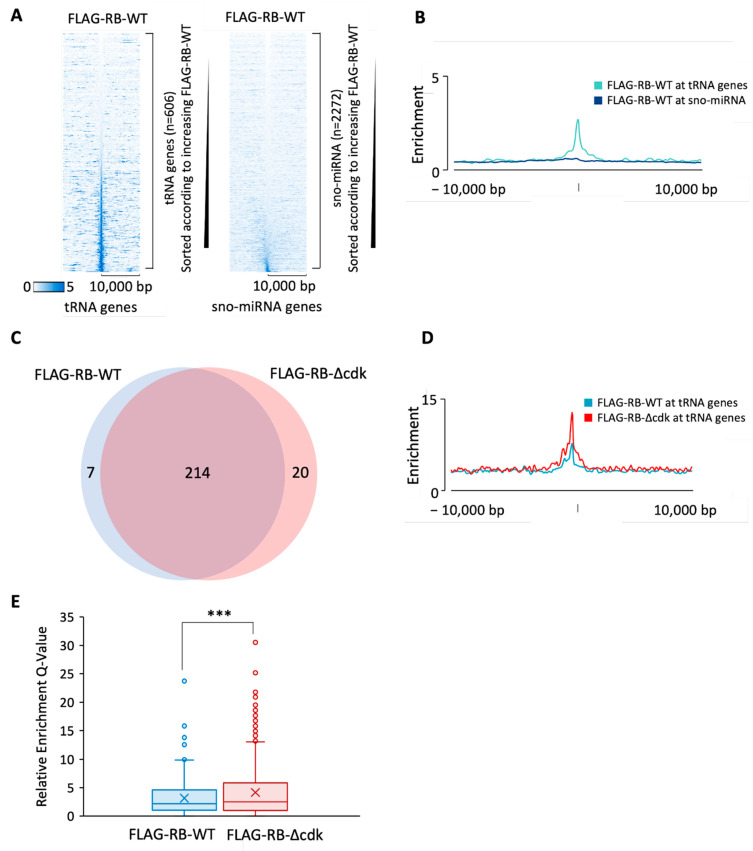
Substitution of CDK phosphoacceptor sites increases binding of RB to many tRNA genes. (**A**) Heatmap depicting RB binding 10 kb either side of hg19 tRNA genes (left) or sno/miRNA genes (right) in RPE1 cells. (**B**) Average signal intensity of RB binding at tRNA genes (green) or sno/miRNA genes (dark blue) in RPE1 cells. (**C**) Overlap in WT-RB and Δcdk-RB binding at tRNA genes. (**D**) Average signal intensity of FLAG-RB-WT (blue) and FLAG-RB-Δcdk (red) binding at tRNA genes in RPE1 cells. (**E**) Box plot depicting the average binding enrichment of WT-RB or Δcdk-RB mutant at tRNA genes. Boxes show the median (solid line) ± one quartile, with the mean denoted by a cross; whiskers extend to the furthest data point within a 1.5× interquartile range. Unpaired, two-tailed Student’s *t*-tests were applied to calculate statistical significance. *** denotes *p* < 0.0001.

Sanidas et al. adopted the same approach in RPE1 human retinal pigment epithelial cells, replacing the endogenous RB with similar levels of flag-tagged wild-type or RBΔcdk mutant [37,101]. Use of retinal epithelial cells is particularly apposite as inherited mutations in the *RB1* gene cause oncogenic transformation of this cell type early in life [1]. As in fibroblasts, RB binds selectively to many tRNA genes in RPE1 cells but is only detected at a small minority of miRNA and snoRNA genes (Figure 5A,B). Nearly all tRNA genes bound by wild-type RB are also bound by the RB∆cdk mutant (Figure 5C). However, occupancy of tDNA loci is, in many cases, strengthened significantly by the phosphoresistant mutations (Figure 5D,E).

These data suggest that CDK-mediated phosphorylation of RB can regulate its recruitment to tRNA genes in both BJ and RPE1 cells. However, it cannot be discounted that the alanine substitutions in this mutant have influenced interactions through mechanisms additional to their exclusion of phosphorylation.

### 3.3. RB Recruitment to Many tRNA Genes Coincides with Binding Sites for E2F

**Figure 6:**
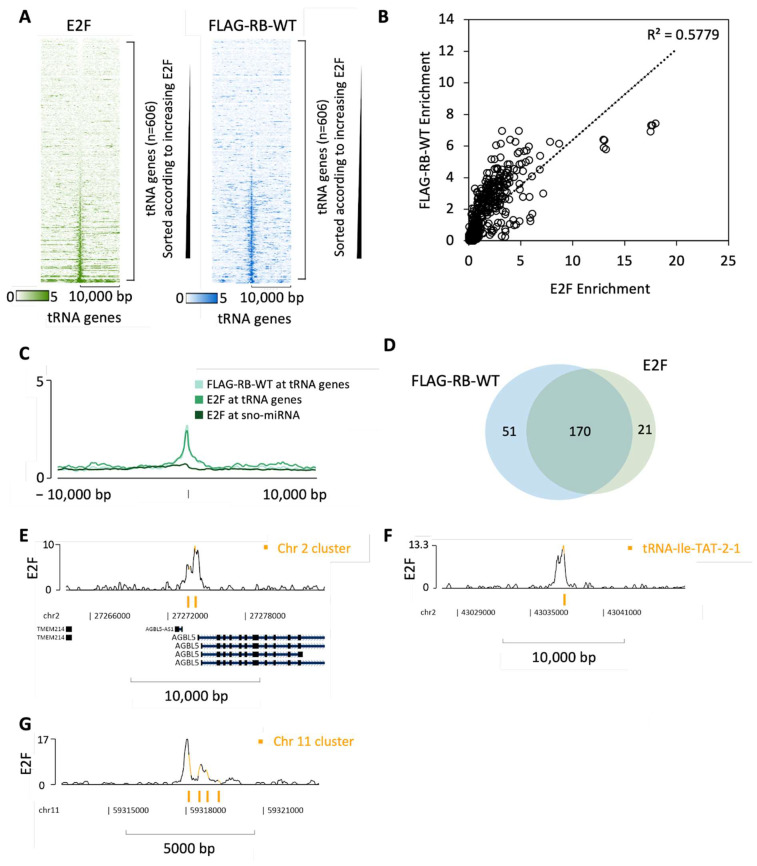
Binding of RB overlaps with E2F1 at tRNA genes in RPE1 cells. (**A**) Heatmap depicting E2F1 (green) or FLAG-RB-WT (blue) binding 10 kb either side of hg19 tRNA genes sorted according to increasing E2F1 in RPE1 cells. (**B**) Correlation plot of RB and E2F1 enrichment (Q-values) at tRNA genes. The Pearson coefficient of determination R^2^ is 0.5779. (**C**) Average signal intensity of E2F1 (green), and RB (light blue) binding at tRNA genes or E2F1 binding at sno/miRNA genes (dark green) in RPE1 cells. (**D**) Overlap in tRNA genes bound by RB and E2F. The threshold for binding was set as the mean binding enrichment for each group. (**E**–**G**) Representative line tracks showing E2F binding at tRNA-Tyr-GTA-2-1 and tRNA-Ala-AGC-8-1 (**E**), tRNA-Ile-TAT-2-1 (**F**) and a cluster of four tRNA genes on chromosome 11 (**G**). tRNA genes are highlighted in orange.

RB lacks a DNA-binding domain and is recruited to genomic sites through interaction with transcription factors that recognize specific DNA sequences, the best-documented of which is E2F. Although tRNA genes have not, to our knowledge, been reported as E2F targets, ChIP-seq revealed that ~190 are bound by E2F1 in RPE1 cells; furthermore, a heatmap with RB occupancy sorted according to the strength of E2F1 binding demonstrates strong correlation at tRNA loci (Figure 6A). Indeed, RB is recruited to most of the tRNA genes that are close to sites bound by E2F1, whereas minimal enrichment of either was detected at the majority of miRNA and snoRNA genes (Figure 6B–D). In some cases, both the tRNA gene(s) and the E2F1 site(s) are located within the promoter region of a protein-coding gene; for example, the binding of RB and E2F1 coincide with the tRNA-Tyr-GTA-2-1 and tRNA-Ala-AGC-8-1 genes upstream of the AGBL5 gene on chromosome 2 (Figure 6E). However, E2F1 and RB can also be found at tRNA genes located far from protein-coding genes, such as the isolated tRNA-Ile-TAT-2-1 gene on chromosome 2 (Figure 6F) and a cluster of four tRNA genes on chromosome 11 (Figure 6G). Mining of public ChIP-seq data using ChIP-Atlas (http://chip-atlas.org) [104,105] reveals an additional 12 cell lines in which E2F1 has been detected at the tRNA-Ile-TAT-2-1 gene (Supplementary Figure S3). The well-established ability of DNA-bound E2F1 to recruit RB to its target sites offers an explanation for the detection of RB at many tRNA genes.

**Figure 7:**
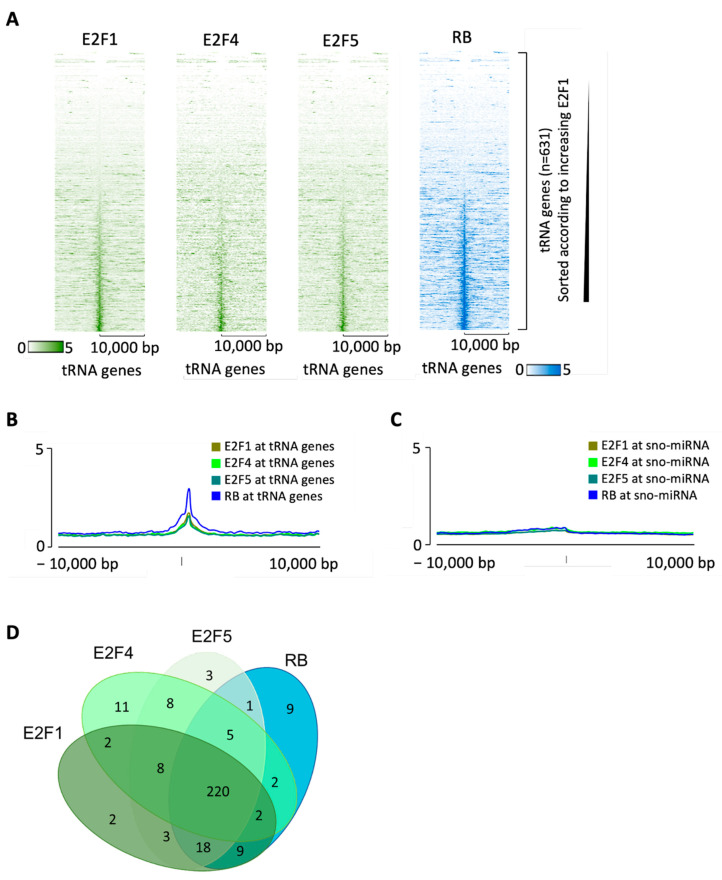
Binding of RB overlaps with E2F at tRNA genes in K562 cells. (**A**) Heatmap depicting E2F1, E2F4 or E2F5 (green) or RB (blue) binding 10 kb either side of hg38 tRNA genes, sorted according to increasing E2F1 in K562 cells. (**B**,**C**) Average signal intensity of E2F1, E2F4, E2F5 and RB binding at tRNA genes (**B**) or sno-miRNA genes (**C**) in K562 cells. (**D**) Overlap in tRNA genes bound by RB, E2F1, E2F4 and E2F5 in K562 cells. The threshold for binding was set as the mean binding enrichment for each group.

E2F1 is expressed primarily in proliferating cells and does not accumulate in quiescent cells, where E2F4 and E2F5 are the most abundant members of the E2F family [12]. If tDNA occupancy by RB in quiescent cells (Figure 3) is also mediated by E2F, then E2F4 and/or E2F5 are likely to substitute for E2F1. The encyclopedia of DNA elements (ENCODE) contains ChIP-seq datasets for RB, E2F1, E2F4 and E2F5 in the erythroid cell line K562 (available at www.encodeproject.org/), which reveal that each of these proteins can be found at many tDNAs (Figure 7A,B). Comparable enrichment is not detected at the majority of miRNA and snoRNA genes (Figure 7C). There is substantial overlap between the tDNAs bound by these four proteins (Figure 7D). This is also evident from Figure 7A, where the tDNAs are sorted in each heatmap according to the strength of E2F1 binding; it is evident that the orders of binding of E2F4, E2F5 and RB closely follow that of E2F1. Under these conditions, ~30% of tDNAs are occupied above threshold by at least one of the three E2F family members tested and binding by RB is detected at 87% of these. Of the 266 tDNAs that recruit RB above threshold under these conditions, ~97% bind one or more of E2F1, E2F4 and E2F5. These data suggest that E2F may be primarily responsible for the association of RB with tRNA genes, although alternative recruitment mechanisms are also likely in at least some cases.

**Figure 8:**
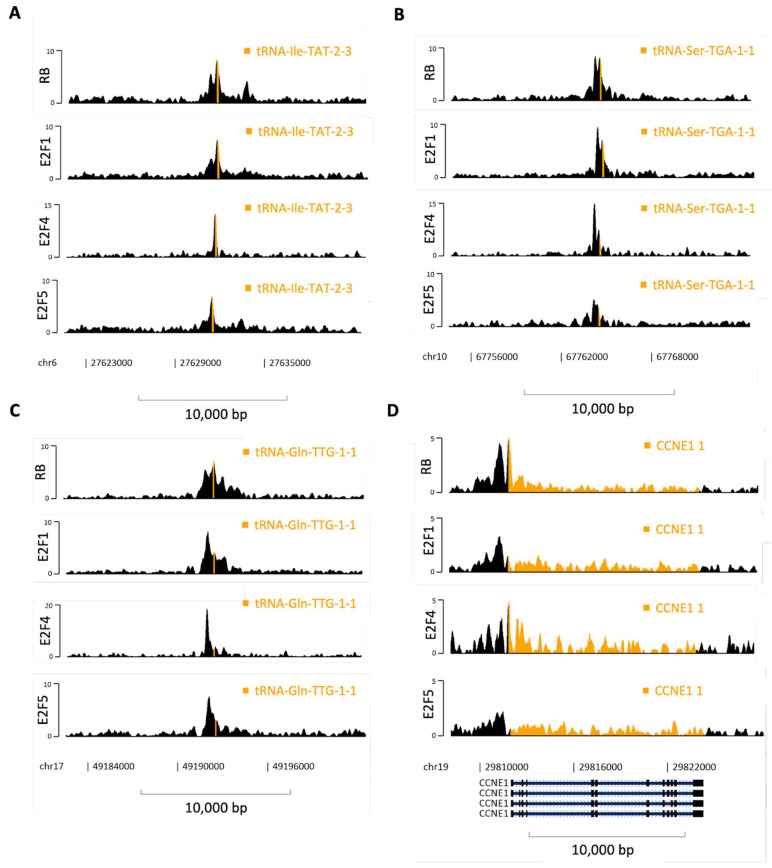
Binding of RB, E2F1, E2F4 and E2F5 at various genes. (**A**–**D**) Representative fill tracks showing RB, E2F1, E2F4 and E2F5 binding at hg38 tRNA-Ile-TAT-2-3 (**A**), tRNA-Ser-TGA-1-1 (**B**), tRNA-Gln-TGG-1-1 (**C**) and cyclin E (CCNE1 (**D**)) genes in K562 cells. Genes are highlighted in orange. Note different y-axis scales between genes and proteins.

Figure 8 shows three examples of isolated tDNAs, far removed from protein-coding genes, where RB, E2F1, E2F4 and E2F5 can all be seen to bind robustly in K562 cells, with well-resolved ChIP-seq peaks that are clearly distinguishable from the surrounding regions (Figure 8A–C). For comparison, Figure 8D shows binding at the cyclin E promoter, a paradigm target for RB and E2F [12,13,31,32,106]. It is noteworthy that the peaks for RB, E2F1, E2F4 and E2F5 at the cyclin E promoter are all weaker than those seen for these proteins in the same datasets at the tRNA genes shown in Figure 8A–C (note different scales). For example, binding of E2F4 has a Q-value of ~5 at the cyclin E promoter, but Q-values of 15–20 are reached at the three tRNA loci. Although these tDNAs were selected as robust examples, they illustrate clearly that some pol III-transcribed genes can recruit RB and E2F at least as efficiently as a well-established target promoter of a gene that is central to cell cycle control. The data provide evidence that E2F family members occupy chromatin sites in close proximity to many tRNA genes, providing a likely explanation for the observed recruitment of RB.

### 3.4. RB May Be Recruited by E2F to 7SL and RMRP Genes

**Figure 9:**
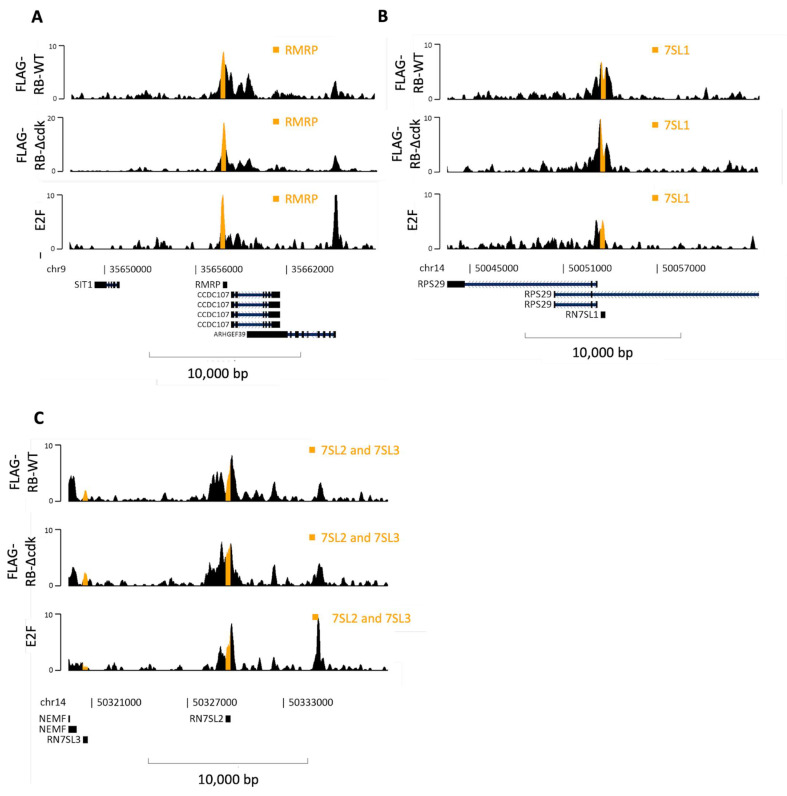
Binding of FLAG-RB-WT, FLAG-RB-Δcdk and E2F1 at other pol III-transcribed genes. (**A**–**C**) Representative line tracks showing FLAG-RB-WT, FLAG-RB-Δcdk and E2F1 binding at hg38 *RMRP* (**A**), *RN7SL1* (**B**), *RN7SL2* and *RN7SL3* (**C**) genes in K562 cells. Genes are highlighted in orange. Note different y-axis scale for FLAG-RB-Δcdk at *RMRP*.

We extended our analyses to include the *RMRP* and *7SL* RNA genes that require pol III for their transcription because they are implicated in carcinogenesis. RMRP is a non-coding RNA of 267 nucleotides that promotes cell cycle progression and proliferation [82,83,85]. It contributes to the processing of pre-rRNA and also associates with the catalytic subunit of telomerase to form an RNA-dependent RNA polymerase [83,107,108,109,110]. Germline loss of the *RMRP* gene can cause an inherited syndrome involving compromised growth [111,112]. The *RMRP* gene undergoes focal amplification in several tumor types and somatic mutations in its promoter lead to elevated expression in breast cancers [84,86]. A peak of E2F1 occupancy occurs at the *RMRP* gene in RPE1 retinal cells and this co-localizes with a site of RB recruitment (Figure 9A). The RB∆cdk mutant is recruited more strongly than wild-type RB, consistent with the ability of CDK-mediated phosphorylation to inhibit its binding to E2F [30]. ChIP-Atlas shows that the RMRP gene is bound by E2F1 in ten additional cell types besides RPE1 and is also bound by E2F4 and E2F5 in K562 cells (Supplementary Figure S4). In addition, this gene is bound in IMR90 cells by p107, which interacts preferentially with E2F4 [12].

7SL is a 300 nucleotide non-coding RNA scaffold for the signal recognition particle which directs nascent secreted proteins to the endoplasmic reticulum [113]. Analysis of multiple specimens from 19 types of cancer revealed consistently elevated expression of 7SL RNA relative to healthy tissue from the same patients [114]. This may impact p53 expression, as translation of p53 mRNA can be suppressed by its hybridization to 7SL RNA [115]. This non-coding RNA has also been demonstrated to promote inflammatory responses in breast cancer; after transmission in exosomes from stromal to malignant cells, 7SL RNA activates the RIG-1 pattern recognition receptor, which induces immune cell infiltration and influences tumor growth and metastasis, as well as therapy resistance [81]. Clear peaks of RB that coincide with E2F1 can be found at the *RN7SL1* and *RN7SL2* genes in RPE1 cells, whereas binding at the *RN7SL3* gene is close to background (Figure 9B,C). RB and E2F1 are also found at *RN7SL1* and *RN7SL2* in K562 cells, where E2F4 and E2F5 also bind *RN7SL2* (Supplementary Figure S5). In contrast, RB is not detected at the *RN7SL3* gene in any of the ChIP-Atlas datasets, despite being bound by E2F1 in MCF7 and HMEC breast cells. These observations provide further evidence that the recruitment of RB to pol III-transcribed genes is selective, in contrast to the general repression mediated by its binding to TFIIIB.

## 4. Discussion

It has been well documented that RB can repress the pol III-mediated synthesis of non-coding RNAs both in vitro and in vivo [48]. This control has been attributed to its interaction with TFIIIB, the ubiquitous factor responsible for recruiting pol III to all of its transcription templates, although additional interactions with ancillary factors TFIIIC and SNAPc have also been reported [39,40,41,42,43,44,45]. Our analysis of multiple ChIP-seq datasets provides evidence that RB may, in addition, act on subsets of pol III-dependent genes through its recruitment by DNA-bound E2F, thereby supplementing the control exerted through TFIIIB. This hypothesis is based on extensive correlative data, demonstrating that RB and E2F occupy overlapping sites at hundreds of tRNA genes in multiple cell types and that the strength of RB binding at these sites correlates with that of E2F. Given the abundant evidence that E2F binds directly to RB and recruits it to genomic sites, it seems likely that this is also the case at pol III-transcribed genes. However, definitive proof will require experimental manipulation to establish if E2F is indeed responsible for the RB recruitment identified here. It is likely that other transcription factors besides E2F contribute in at least some cases, given the large number that have been demonstrated to bind to RB [35,36].

**Figure 10:**
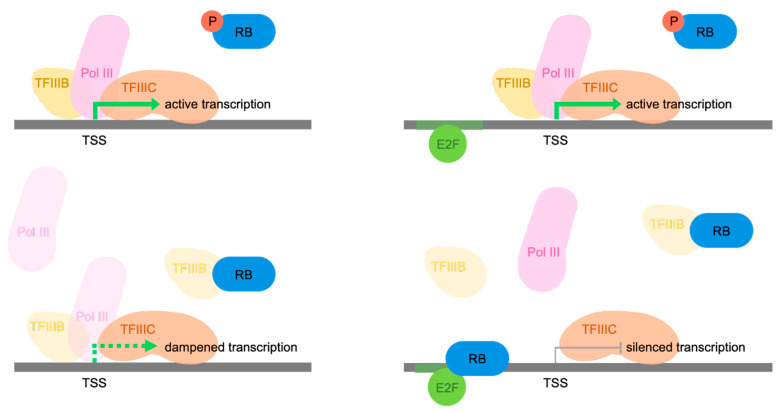
Schematic representation of revised model of Pol III repression by RB. When dephosphorylated, RB can associate with TFIIIB, blocking its interaction with TFIIIC and pol III to preclude formation of the preinitiation complex. In this way, RB can dampen transcription of all pol III-dependent genes with internal promoters (**bottom left**). However, RB can also be tethered to the promoters of a subset of these genes by E2F (**bottom right**), providing potential for additional gene-targeted control. These interactions are released when RB is phosphorylated (**top**).

We speculate that E2F-mediated control could provide much greater regulatory flexibility than repression through TFIIIB. As all pol III transcription requires TFIIIB, its interaction with RB is expected to dampen the expression of all templates, although weaker promoters may be more readily inhibited than strong ones. In contrast, E2F binding is only detected at subsets of pol III-transcribed genes, providing potential for gene-selective control. When tethered to specific promoters by E2F, RB can recruit epigenetic regulators that establish chromatin states that are refractory to transcription [15,16,17,18,19,20,21,22,23,24,25,26,27,28,29]. Epigenetic repressors recruited by RB include histone deacetylases and the H3K9 methylase SUV39H1 [19,20,24,25], which have been shown to inhibit transcription by pol III [91,116]. We envisage that distinct levels of control may be mediated by RB: (1) a restraining effect on the production of all pol III products through interactions with TFIIIB that prevent its recruitment to promoters, as originally suggested [91]; and (2) the selective repression of subsets of pol III-transcribed genes with nearby binding sites for E2F, which may then be subject to epigenetic silencing via locally tethered RB (Figure 10). The interactions with TFIIIB offer a mechanism by which to restrict cell growth by reducing the availability of the essential components of cells’ biosynthetic machinery, including tRNA, 5S rRNA and 7SL RNA. In contrast, selective control of individual genes via E2F may allow cells to fine-tune and optimize relative levels of specific products according to circumstances.

Many studies have demonstrated that relative changes in the expression or activity of a particular tRNA can result in codon-biased reprogramming of the translatome such that mRNAs enriched in a cognate codon are differentially regulated [51,54,70,76,117,118]. As well as selectively adjusting relative rates of translation, the abundance of a tRNA can also impact the stability of mRNAs enriched in its cognate codon(s) [70,119]. Although the synthesis of most tRNAs is elevated in proliferating cells, a subset is down-regulated under these conditions and instead becomes induced during cell differentiation; the anticodons of these tRNAs match codons that are enriched in mRNAs induced under the same conditions [120]. Similarly, many of the mRNAs and tRNAs induced most strongly during growth and proliferation display codon/anticodon correlations consistent with optimization of translational efficiency [120].

Much is now known about mechanisms controlling the overall output of pol III, such as the interaction of TFIIIB with RB in resting cells and with MYC in growing cells [48,51], but mechanisms contributing to the differential regulation of tRNA genes have yet to be dissected in most cases [121]. SOX4 provides one of the few examples identified to date, as it was found, when overexpressed in glioblastoma cells, to bind 126 tRNA genes and diminish their ability to recruit TFIIIB and hence pol III, resulting in selective repression [122]. Our discovery of E2F, RB and p130 binding in close proximity to subsets of tRNA genes suggests additional ways in which differential control may be achieved. For example, the binding of RB above the threshold is only detected at 29% of tRNA genes in quiescent IMR90 cells. Furthermore, despite the considerable overlap between tRNA genes occupied by RB in growing, quiescent and senescent IMR90 cells, there are also subsets of targets that are only bound above the threshold under one or two of these conditions (Figure 3D).

E2F4 and E2F5 are classified as “repressive” E2Fs and are considered of particular importance for inducing cell cycle exit and terminal differentiation [12]. In contrast, E2F1 is classified as “activating” and has a region that is capable of stimulating transcription when released from binding to RB after the G1/S transition [12]. This raises the question of whether it may stimulate expression of pol III products during S and G2 phases, when transcription by pol III is maximal [123]. In the case of canonical pol II-dependent target genes, E2F1 was shown to activate transcription through interactions with TFIIA and TAF subunits of TFIID [23]. As these are not used by tRNA genes, the activation domain of E2F1 may have little or no influence in this context, other than as a means to recruit RB. However, interactions with components of the pol III machinery remain a possibility.

Genes involved in DNA replication and cell cycle regulation, as well as repair of DNA damage, are highly enriched amongst genomic E2F targets [13]. In addition, RB can also be recruited by E2F to many genes involved in RNA metabolism and ribosome biogenesis [101]. Co-regulation of such genes with pol III transcription is consistent with the peak of pol III output in S and G2 phases [43,123] and the essential roles in ribosome assembly and function of 5S rRNA, tRNA and 7SL RNA, the most abundant pol III products.

## 5. Conclusions

Analysis of ChIP-seq data has revealed the presence of RB at a subset of pol III-transcribed genes in a variety of human cell types, including fibroblasts and retinal epithelial cells. This localization is contrary to predictions based on the prevailing model of pol III transcriptional repression by RB. An explanation is offered via the additional discovery that most of these RB-binding sites overlap with positions occupied in the same cells by E2F, the factor most widely associated with the tethering of RB to specific loci. E2F has not previously been linked with large numbers of pol III-dependent genes. The three most prominent members of the E2F family were all found in close proximity to many tRNA genes, with substantial overlap in the sites targeted, but also examples that are occupied more selectively by individual family members. We propose a model in which RB operates at two levels to influence the synthesis of non-coding RNAs by pol III: (1) general dampening of all output as a restraining influence on cell growth; (2) differential tethering to subsets of genes to achieve more discriminatory effects. Loss of such controls when RB becomes inactivated can be expected to contribute to the overall increase in expression of pol III products observed in most cancers, which, in certain cases, may promote cancer progression.

## Data Availability

Data are contained within the article and Supplementary Materials.

## Supplementary Materials

The following supporting information can be downloaded at https://www.mdpi.com/article/10.3390/cancers16030481/s1: Figure S1: p107 associates specifically with a subset of tRNA genes in IMR90 cells. Figure S2: Most tRNA genes that associate with RB above threshold are common between BJ and IMR90 lines of human diploid fibroblasts. Figure S3: ChIP-Atlas peak-call data for RB and E2F factors at a tRNA gene. Figure S4: ChIP-Atlas peak-call data for RB and E2F factors at the RMRP gene. Figure S5: ChIP-Atlas peak-call data for RB and E2F factors at two 7SL genes.

## References

[1] Friend S.H., Bernards R., Rogelj S., Weinberg R.A., Rapaport J.M., Alberts D.M., Dryja T.P. (1986). A human DNA segment with properties of the gene that predisposes to retinoblastoma and osteosarcoma. Nature.

[2] Weinberg R.A. (1995). The retinoblastoma protein and cell cycle control. Cell.

[3] Whyte P. (1995). The retinoblastoma protein and its relatives. Semin. Cancer Biol..

[4] Jacks T., Fazeli A., Schmitt E.M., Bronson R.T., Goodell M.A., Weinberg R.A. (1992). Effects of an Rb mutation in the mouse. Nature.

[5] Lee E.Y.-H.P., Chang C.-Y., Hu N., Wang Y.-C.J., Lai C.-C., Herrup K., Lee W.-H., Bradley A. (1992). Mice deficient for Rb are nonviable and show defects in neurogenesis and haematopoiesis. Nature.

[6] Hu N., Gutsmann A., Herbert D.C., Bradley A., Lee W.H., Lee E.Y. (1994). Heterozygous Rb-1^D20/+^ mice are predisposed to tumours of the pituitary gland with a nearly complete penetrance. Oncogene.

[7] Maandag E., van der Valk M., Vlaar M., Feltkamp C., O’Brien J., van Roon M., van der Lugt N., Berns A., Riele H.T. (1994). Developmental rescue of an embryonic-lethal mutation in the retinoblastoma gene in chimeric mice. EMBO J..

[8] Williams B., Schmitt E., Remington L., Bronson R., Albert D., Weinberg R., Jacks T. (1994). Extensive contribution of Rb-deficient cells to adult chimeric mice with limited histopathological consequences. EMBO J..

[9] Huang H.-J.S., Yee J.-K., Shew J.-Y., Chen P.-L., Bookstein R., Friedmann T., Lee E.Y.-H.P., Lee W.-H. (1988). Suppression of the neoplastic phenotype by replacement of the RB gene in human cancer cells. Science.

[10] Bookstein R., Shew J., Chen P., Scully P., Lee W. (1990). Suppression of tumorigenicity of human prostate carcinoma cells by replacing a mutated RB gene. Science.

[11] Qin X., Chittenden T., Livingston D.M., Kaelin W.G. (1992). Identification of a growth suppression domain within the retinoblastoma gene product. Genes Dev..

[12] Trimarchi J.M., Lees J.A. (2001). Sibling rivalry in the E2F family. Nat. Rev. Mol. Cell Biol..

[13] Braken A.P., Ciro M., Cocito A., Helin K. (2004). E2F target genes: Unraveling the biology. Trends Biochem. Sci..

[14] Xie D., Pei Q., Li J., Wan X., Ye T. (2021). Emerging Role of E2F Family in Cancer Stem Cells. Front. Oncol..

[15] Weintraub S.J., Prater C.A., Dean D.C. (1992). Retinoblastoma protein switches the E2F site from positive to negative element. Nature.

[16] Flemington E.K., Speck S.H., Kaelin W.G.J. (1993). E2F-1-mediated transactivation is inhibited by complex formation with the retinoblastoma susceptibility gene product. Proc. Natl. Acad. Sci. USA.

[17] Helin K., Harlow E., Fattaey A. (1993). Inhibition of E2F-1 transactivation by direct binding of the retinoblastoma protein. Mol. Cell Biol..

[18] Weintraub S.J., Chow K.N.B., Luo R.X., Zhang S.H., He S., Dean D.C. (1995). Mechanism of active transcriptional repression by the retinoblastoma protein. Nature.

[19] Brehm A., Miska E.A., McCance D.J., Reid J.L., Bannister A.J., Kouzarides T. (1998). Retinoblastoma protein recruits histone deacetylase to repress transcription. Nature.

[20] Luo R.X., Postigo A.A., Dean D.C. (1998). Rb interacts with histone deacetylase to repress transcription. Cell.

[21] Magnaghi-Jaulin L., Groisman R., Naguibneva I., Robin P., Lorain S., Le Villain J.P., Troalen F., Trouche D., Harel-Bellan A. (1998). Retinoblastoma protein represses transcription by recruiting a histone deacetylase. Nature.

[22] Brehm A., Kouzarides T. (1999). Retinoblastoma protein meets chromatin. Trends Biochem. Sci..

[23] Ross J.F., Liu X., Dynlacht B.D. (1999). Mechanism of transcriptional repression of E2F by the retinoblastoma tumour suppressor protein. Mol. Cell.

[24] Robertson K.D., Ait-Si-Ali S., Yokochi T., Wade P.A., Jones P.L., Wolffe A.P. (2000). DNMT1 forms a complex with Rb, E2F1, and HDAC1 and represses transcription from E2F-responsive promoters. Nat. Genet..

[25] Nielsen S.J., Schneider R., Bauer U.M., Bannister A.J., Morrison A., O’Carroll D., Firestein R., Cleary M., Jenuwein T., Herrera R.E. (2001). Rb targets histone H3 methylation and HP1 to promoters. Nature.

[26] Vandel L., Nicolas E., Vaute O., Ferreira R., Ait-Si-Ali S., Trouche D. (2001). Transcriptional Repression by the Retinoblastoma Protein through the Recruitment of a Histone Methyltransferase. Mol. Cell Biol..

[27] Pradhan S., Kim G.D. (2002). The retinoblastoma gene product interacts with maintenance human DNA (cytosine-5) methyltransferase and modulates its activity. EMBO J..

[28] Ogawa H., Ishiguro K., Gaubatz S., Livingston D.M., Nakatani Y. (2002). A Complex With Chromatin Modifiers That Occupies E2F- and Myc-Responsive Genes in G0 Cells. Science.

[29] Selvakumar T., Gjidoda A., Hovde S.L., Henry R.W. (2012). Regulation of human RNA polymerase III transcription by DNMT1 and DNMT3a DNA methyltransferases. J. Biol. Chem..

[30] Adams P.D., Kaelin W.G. (1995). Transcriptional control by E2F. Semin. Cancer Biol..

[31] Herrera R.E., Sah V.P., Williams B.O., Makela T.P., Weinberg R.A., Jacks T. (1996). Altered cell cycle kinetics, gene expression, and G_1_ restriction point regulation in Rb-deficient fibroblasts. Mol. Cell Biol..

[32] Hurford R.K., Cobrinik D., Lee M.-H., Dyson N. (1997). pRB and p107/p130 are required for the regulated expression of different sets of E2F responsive genes. Genes Dev..

[33] Pines J. (1995). Cyclins, CDKs and cancer. Semin. Cancer Biol..

[34] Mittnacht S. (1998). Control of pRB phosphorylation. Curr. Opin. Genet. Dev..

[35] Mulligan G., Jacks T. (1998). The retinoblastoma gene family: Cousins with overlapping interests. Trends Genet..

[36] Morris E.J., Dyson N.J. (2001). Retinoblastoma protein partners. Adv. Cancer Res..

[37] Sanidas I., Morris R., Fella K.A., Rumde P.H., Boukhali M., Tai E.C., Ting D.T., Lawrence M.S., Haas W., Dyson N.J. (2019). A code of mono-phosphorylation modulates the function of RB. Mol. Cell.

[38] Schramm L., Hernandez N. (2002). Recruitment of RNA polymerase III to its target promoters. Genes Dev..

[39] Larminie C.G.C., Cairns C.A., Mital R., Martin K., Kouzarides T., Jackson S.P., White R.J. (1997). Mechanistic analysis of RNA polymerase III regulation by the retinoblastoma protein. EMBO J..

[40] Chu W.-M., Wang Z., Roeder R.G., Schmid C.W. (1997). RNA polymerase III transcription repressed by Rb through its interactions with TFIIIB and TFIIIC2. J. Biol. Chem..

[41] Larminie C.G.C., Sutcliffe J.E., Tosh K., Winter A.G., Felton-Edkins Z.A., White R.J. (1999). Activation of RNA polymerase III transcription in cells transformed by simian virus 40. Mol. Cell Biol..

[42] Sutcliffe J.E., Cairns C.A., McLees A., Allison S.J., Tosh K., White R.J. (1999). RNA polymerase III transcription factor IIIB is a target for repression by pocket proteins p107 and p130. Mol. Cell Biol..

[43] Scott P.H., Cairns C.A., Sutcliffe J.E., Alzuherri H.M., Mclees A., Winter A.G., White R.J. (2001). Regulation of RNA polymerase III transcription during cell cycle entry. J. Biol. Chem..

[44] Felton-Edkins Z.A., White R.J. (2002). Multiple mechanisms contribute to the activation of RNA polymerase III transcription in cells transformed by papovaviruses. J. Biol. Chem..

[45] Hirsch H.A., Jawdekar G.W., Lee K.-A., Gu L., Henry R.W. (2004). Distinct mechanisms for repression of RNA polymerase III transcription by the retinoblastoma tumor suppressor protein. Mol. Cell Biol..

[46] Angelis E., Garcia A., Chan S.S., Schenke-Layland K., Ren S., Goodfellow S.J., Jordan M.C., Roos K.P., White R.J., MacLellan W.R. (2008). A cyclin D2-Rb pathway regulates cardiac myocyte size and RNA polymerase III after biomechanical stress in adult myocardium. Circ. Res..

[47] Dieci G., Fiorino G., Castelnuovo M., Teichmann M., Pagano A. (2007). The expanding RNA polymerase III transcriptome. Trends Genet..

[48] White R.J. (2005). RNA polymerases I and III, growth control and cancer. Nat. Rev. Mol. Cell Biol..

[49] Grewal S.S. (2015). Why should cancer biologists care about tRNAs? tRNA synthesis, mRNA translation and the control of growth. Biochem. Biophys. Acta.

[50] Huang S., Sun B., Xiong Z., Shu Y., Zhou H., Zhang W., Xiong J., Li Q. (2018). The dysregulation of tRNAs and tRNA derivatives in cancer. J. Exp. Clin. Cancer Res..

[51] Orellana E.A., Siegal E., Gregory R.I. (2022). tRNA dysregulation and disease. Nat. Rev. Genet..

[52] Sangha A.K., Kantidakis T. (2022). The aminoacyl-tRNA synthetase and tRNA expression levels are deregulated in cancer and correlate independently with patient survival. Curr. Issues Mol. Biol..

[53] Vincent C.T., Schneider R.J. (2022). Selective tRNA charging in breast cancer. Nat. Cell Biol..

[54] Pinzaru A.M., Tavazoie S.F. (2023). Transfer RNAs as dynamic and critical regulators of cancer progression *Nat*. Rev. Cancer.

[55] Hirsch H.A., Gu L., Henry R.W. (2000). The retinoblastoma tumor suppressor protein targets distinct general transcription factors to regulate RNA polymerase III gene expression. Mol. Cell Biol..

[56] White R.J., Trouche D., Martin K., Jackson S.P., Kouzarides T. (1996). Repression of RNA polymerase III transcription by the retinoblastoma protein. Nature.

[57] Brown T.R.P., Scott P.H., Stein T., Winter A.G., White R.J. (2000). RNA polymerase III transcription: Its control by tumor suppressors and its deregulation by transforming agents. Gene Expr..

[58] Felton-Edkins Z.A., Kenneth N.S., Brown T.R.P., Daly N.L., Gomez-Roman N., Grandori C., Eisenman R.N., White R.J. (2003). Direct regulation of RNA polymerase III transcription by RB, p53 and c-Myc. Cell Cycle.

[59] Grana X., Garriga J., Mayol X. (1998). Role of the retinoblastoma protein family, pRB, p107 and p130 in the negative control of cell growth. Oncogene.

[60] Claudio P.P., Howard C.M., Baldi A., De Luca A., Fu Y., Condorelli G., Sun Y., Colburn N., Calabretta B., Giordano A. (1994). p130/Rb2 has growth suppressive properties similar to yet distinctive from those of retinoblastoma family members pRb and p107. Cancer Res..

[61] Woo M.S.-A., Sanchez I., Dynlacht B.D. (1997). p130 and p107 use a conserved domain to inhibit cellular cyclin-dependent kinase activity. Mol. Cell Biol..

[62] Zhu L., van den Heuvel S., Helin K., Fattaey A., Ewen M., Livingston D., Dyson N., Harlow E. (1993). Inhibition of cell proliferation by p107, a relative of the retinoblastoma protein. Genes Dev..

[63] Cobrinik D., Lee M.H., Hannon G., Mulligan G., Bronson R.T., Dyson N., Harlow E., Beach D., Weinberg R.A., Jacks T. (1996). Shared role of the pRB-related p130 and p107 proteins in limb development. Genes Dev..

[64] Clarke A.R., Maandag E.R., van Roon M., van der Lugt N.M.T., van der Valk M., Hooper M.L., Berns A., te Riele H. (1992). Requirement for a functional Rb-1 gene in murine development. Nature.

[65] Burkhart D.L., Sage J. (2008). Cellular mechanisms of tumour suppression by the retinoblastoma gene. Nat. Rev. Cancer.

[66] Nasmyth K. (1996). Another role rolls in. Nature.

[67] White R.J. (1997). Regulation of RNA polymerases I and III by the retinoblastoma protein: A mechanism for growth control?. Trends Biochem. Sci..

[68] Rideout E.J., Marshall L., Grewal S.S. (2012). Drosophila RNA polymerase III repressor Maf1 controls body size and developmental timing by modulating tRNAiMet synthesis and systemic insulin signaling. Proc. Natl. Acad. Sci. USA.

[69] Pavon-Eternod M., Gomes S., Rosner M.R., Pan T. (2013). Overexpression of initiator methionine tRNA leads to global reprogramming of tRNA expression and increased proliferation in human epithelial cells. RNA.

[70] Goodarzi H., Nguyen H.C.B., Zhang S., Dill B.D., Molina H., Tavazoie S.F. (2016). Modulated expression of specific tRNAs drives gene expression and cancer progression. Cell.

[71] Birch J., Clarke C.J., Campbell A.D., Campbell K., Mitchell L.E., Liko D., Kalna G., Strathdee D., Sansom O.J., Neilson M. (2016). The initiator methionine tRNA drives cell migration and invasion leading to increased metastatic potential in melanoma. Biol. Open.

[72] Clarke C.J., Berg T.J., Birch J., Ennis D., Mitchell L.E., Cloix C., Campbell A.D., Sumpton D., Nixon C., Campbell K. (2016). The initiator methionine tRNA drives secretion of type II collagen from stromal fibroblasts to promote tumor growth and angiogenesis *Curr*. Biol..

[73] Macari F., El-houfi Y., Boldina G., Xu H., Khoury-Hanna S., Ollier J., Yazdani L., Zheng G., Bieche I., Legrand N. (2016). TRIM6/61 connects PKCa with translational control through tRNAiMet stabilization: Impact on tumorigenesis. Oncogene.

[74] Wang B., Li D., Kovalchuk I., Apel I.J., Chinnalyan A.M., Woycicki R.K., Cantor C.R., Kovalchuk O. (2018). miR-34a directly targets tRNAiMet precursors and affects cellular proliferation, cell cycle, and apoptosis *Proc*. Natl. Acad. Sci. USA.

[75] Dai Z., Liu H., Liao J., Huang C., Ren X., Zhu W., Zhu S., Peng B., Li S., Lai J. (2021). N7-Methylguanosine tRNA modification enhances oncogenic mRNA translation and promotes intrahepatic cholangiocarcinoma progression. Mol. Cell.

[76] Orellana E.A., Liu Q., Yankova E., Pirouz M., De Braekeleer E., Zhang W., Lim J., Aspris D., Sendinc E., Garyfallos D.A. (2021). METTL1-mediated m7G modification of Arg-TCT tRNA drives oncogenic transformation. Mol. Cell.

[77] Santos M., Fidalgo A., Varanda A.S., Soares A.R., Almeida G.M., Martins D., Mendes N., Oliveira C., Santos M.A.S. (2022). Upregulation of tRNA-Ser-AGA-2-1 promotes malignant behavior in normal bronchial cells. Front. Mol. Biosci..

[78] Krishnan P., Ghosh S., Wang B., Heyns M., Li D., Mackey J.R., Kovalchuk O., Damaraju S. (2016). Genome-wide profiling of transfer RNAs and their role as novel prognostic markers for breast cancer. Sci. Rep..

[79] Zhang Z., Ye Y., Gong J., Ruan H., Liu C.-J., Xiang Y., Cai C., Guo A.-Y., Ling J., Diao L. (2018). Global analysis of tRNA and translation factor expression reveals a dynamic landscape of translational regulation in human cancers. Comm. Biol..

[80] Butterfield S.P., Sizer R.E., Rand E., White R.J. (2023). Selection of tRNA Genes in Human Breast Tumours Varies Substantially between Individuals. Cancers.

[81] Nabet B.Y., Qiu Y., Shabason J.E., Wu T.J., Yoon T., Kim B.C., Benci J.L., DeMichele A.M., Tchou J., Marcotrigiano J. (2017). Exosome RNA unshielding couples stromal activation to pattern recognition receptor signaling in cancer. Cell.

[82] Thiel C.T., Horn D., Zabel B., Ekici A.B., Salinas K., Gebhart E., Ruschendorf F., Sticht H., Spranger J., Muller D. (2005). Severely incapacitating mutations in patients with extreme short stature identify RNA-processing endoribonuclease RMRP as an essential cell growth regulator. Am. J. Hum. Genet..

[83] Goldfarb K.C., Cech T.R. (2017). Targeted CRISPR disruption reveals a role for RNase MRP RNA in human preribosomal RNA processing. Genes Dev..

[84] Rheinbay E., Parasuraman P., Grimsby J., Tiao G., Engreitz J.M., Kim J. (2017). Recurrent and functional regulatory mutations in breast cancer. Nature.

[85] Vakkilainen S., Skoog T., Einarsdottir E., Middleton A., Pekkinen M., Ohman T., Katayama S., Krjutskov K., Kovanen P.E., Varjosalo M. (2019). The human long non-coding RNA gene RMRP has pleiotropic effects and regulates cell-cycle progression at G2. Sci. Rep..

[86] Rheinbay E., Nielsen M.M., Abascal F., Wala J.A., Shapira O., Tiao G., Hornshøj H., Hess J.M., Juul R.I., Lin Z. (2020). Analyses of non-coding somatic drivers in 2,658 cancer whole genomes. Nature.

[87] Geiduschek E.P., Kassavetis G.A. (2001). The RNA polymerase III transcription apparatus. J. Mol. Biol..

[88] Arimbasseri A., Maraia R.J. (2016). RNA polymerase III advances: Structural and tRNA functional views. Trends Biochem. Sci..

[89] Ramsay E.P., Vannini A. (2017). Structural rearrangements of the RNA polymerase III machinery during tRNA transcription initiation. BBA—Gene Regul. Mech..

[90] Kassavetis G.A., Braun B.R., Nguyen L.H., Geiduschek E.P.S. (1990). *S. cerevisiae* TFIIIB is the transcription initiation factor proper of RNA polymerase III, while TFIIIA and TFIIIC are assembly factors. Cell.

[91] Sutcliffe J.E., Brown T.R.P., Allison S.J., Scott P.H., White R.J. (2000). Retinoblastoma protein disrupts interactions required for RNA polymerase III transcription. Mol. Cell Biol..

[92] Gjidoda A., Henry R.W. (2013). RNA polymerase III repression by the retinoblastoma tumor suppressor protein. Biochim. Biophys. Acta.

[93] Schramm L., Pendergrast P.S., Sun Y., Hernandez N. (2000). Different human TFIIIB activities direct RNA polymerase III transcription from TATA-containing and TATA-less promoters. Genes Dev..

[94] Moqtaderi Z., Wang J., Raha D., White R.J., Snyder M., Weng Z., Struhl K. (2010). Genomic binding profiles of functionally distinct RNA polymerase III transcription complexes in human cells. Nat. Struct. Mol. Biol..

[95] Leinonen R., Sugawara H., Shumway M. (2011). The Sequence Read Archive. Nucleic Acids Res..

[96] Langmead B., Salzberg S.L. (2012). Fast gapped-read alignment with Bowtie 2. Nat. Methods.

[97] Press A.R. (2015). Ultrafast and Memory-Efficient Alignment of Short DNA Sequences to the Human Genome.

[98] Consortium T.E.P. (2012). An integrated encyclopedia of DNA elements in the human genome. Nature.

[99] Karolchik D., Hinrichs A.S., Furey T.S., Roskin K.M., Sugnet C.W., Haussler D., Kent W.J. (2004). The UCSC Table Browser data retrieval tool. Nucleic Acids Res..

[100] Lerdrup M., Johansen J.V., Agrawal-Singh S., Hansen K. (2016). An interactive environment for agile analysis and visualization of ChIP-sequencing data. Nat. Struct. Mol. Biol..

[101] Sanidas I., Lee H., Rumde P.H., Boulay G., Morris R., Golczer G., Stanzione M., Hajizadeh S., Zhong J., Ryan M.B. (2022). Chromatin-bound RB targets promoters, enhancers, and CTCF-bound loci and is redistributed by cell-cycle progression. Mol. Cell.

[102] Chicas A., Wang X., Zhang C., McCurrach M., Zhao Z., Mert O., Dickens R.A., Narita M., Zhang M., Lowe S.W. (2010). Dissecting the unique role of the retinoblastoma tumor suppressor during cellular senescence. Cancer Cell.

[103] Ferrari R., Su T., Li B., Bonora G., Oberai A., Chan Y., Sasidharan R., Berk A.J., Pellegrini M., Kurdistani S.K. (2012). Reorganization of the host epigenome by a viral oncogene. Genome Res..

[104] Oki S., Ohta T., Shioi G., Hatanaka H., Ogasawara O., Okuda Y., Kawaji H., Nakaki R., Sese J., Meno C. (2018). ChIP-Atlas: A data-mining suite powered by full integration of public ChIP-seq data. EMBO Rep..

[105] Zou Z., Ohta T., Miura F., Oki S. (2022). ChIP-Atlas 2021 update: A data-mining suite for exploring epigenomic landscapes by fully integrating ChIP-seq, ATAC-seq and Bisulfite-seq data. Nucleic Acids Res..

[106] Wells J., Boyd K.E., Fry C.J., Bartley S.M., Farnham P.J. (2000). Target gene specificity of E2F and pocket protein family members in living cells. Mol. Cell Biol..

[107] Schmitt M.E., Clayton D.A. (1993). Nuclear RNase MRP is required for correct processing of pre-5.8S rRNA in *Saccharomyces cerevisiae*. Mol. Cell Biol..

[108] Clayton D.A. (1994). A nuclear function for RNase MRP. Proc. Natl. Acad. Sci. USA.

[109] Lygerou Z., Allmang C., Tollervey D., Seraphin B. (1996). Accurate processing of a eukaryotic precursor ribosomal RNA by ribonuclease MRP in vitro. Science.

[110] Maida Y., Yasukawa M., Furuuchi M., Lassmann T., Possemato R., Okamoto N., Kasim V., Hayashizaki Y., Hahn W.C., Matsutomi K. (2009). An RNA-dependent RNA polymerase formed by TERT and the RMRP RNA. Nature.

[111] Ridanpaa M., van Eenennaam H., Pelin K., Chadwick R., Johnson C., Yuan B., vanVenrooij W., Pruijn G., Samela R., Rockas S. (2001). Mutations in the RNA component of RNase MRP cause a pleiotropic human disease, cartilage-hair hypoplasia. Cell.

[112] Ridanpaa M., Sistonen P., Rockas S., Rimoin D.L., Makitie O., Kaitila I. (2002). Worldwide mutation spectrum in cartilage-hair hypoplasia: Ancient founder origin of the major 70A->G mutation of the untranslated RMRP. Eur. J. Hum. Genet..

[113] Walter P., Blobel G. (1982). Signal recognition particle contains a 7S RNA essential for protein translocation across the endoplasmic reticulum. Nature.

[114] Chen W., Bocker W., Brosius J., Tiedge H. (1997). Expression of neural BC200 RNA in human tumours. J. Pathol..

[115] Abdelmohsen K., Panda A.C., Kang M.-J., Guo R., Kim J., Grammatikakis I., Yoon J.-H., Dudekula D.B., Noh J.H., Yang X.-L. (2014). 7SL RNA represses p53 translation by competing with HuR. Nucleic Acids Res..

[116] Varshney D., Vavrova-Anderson J., Oler A.J., Cowling V.H., Cairns B.R., White R.J. (2015). SINE transcription by RNA polymerase III is suppressed by histone methylation but not by DNA methylation. Nat. Commun..

[117] Rapino F., Delaunay S., Rambow F., Zhou Z., Tharun L., De Tullio P., Sin O., Shostak K., Schmitz S., Piepers J. (2018). Codon-specific translation reprogramming promotes resistance to targeted therapy. Nature.

[118] Thandapani P., Kloetgen A., Witkowski M.T., Glytsou C., Lee A.K., Wang E., Wang J., LeBoeuf S.E., Avrampou K., Papagiannakopoulos T. (2022). Valine tRNA levels and availability regulate complex I assembly in leukaemia. Nature.

[119] Hanson G., Coller J. (2018). Codon optimality, bias and usage in translation and mRNA decay. Nat. Rev. Mol. Cell Biol..

[120] Gingold H., Tehler D., Christoffersen N.R., Nielsen M.M., Asmar F., Kooistra S.M., Christophersen N.S., Christensen L.L., Borre M., Sorensen K.D. (2014). A dual program for translation regulation in cellular proliferation and differentiation. Cell.

[121] White R.J. (2011). Transcription by RNA polymerase III—More complex than we thought. Nat. Rev. Genet..

[122] Yang J., Smith D.K., Ni H., Wu K.Y., Huang D., Pan S., Sathe A.A., Tang Y., Liu M.-L., Xing C. (2020). SOX4-mediated repression of specific tRNAs inhibits proliferation of human glioblastoma cells. Proc. Natl. Acad. Sci. USA.

[123] White R.J., Gottlieb T.M., Downes C.S., Jackson S.P. (1995). Cell cycle regulation of RNA polymerase III transcription. Mol. Cell Biol..

