# Supplementary material for: Selective Occupation by E2F and RB of Loci Expressed by RNA Polymerase III
Source: Cancers (Basel). 2024 Jan 23;16(3):481. doi: 10.3390/cancers16030481 (PMC10854548; doi:10.3390/cancers16030481)
Supplement: Supplementary file 1 [file cancers-16-00481-s001.zip › cancers-2807351-supplementary.pdf]

Supplementary figures

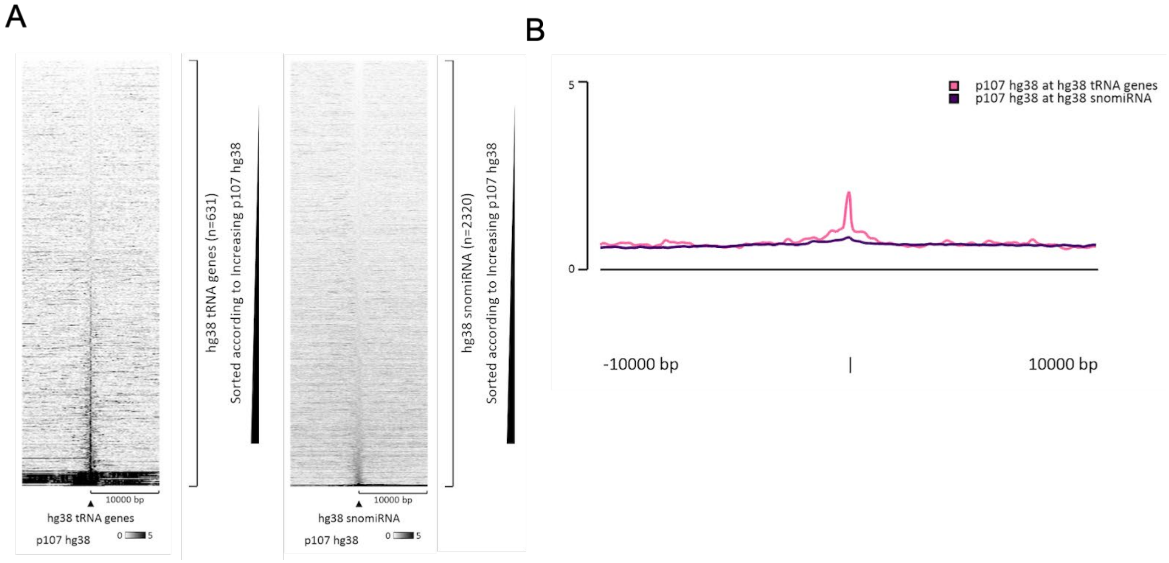

Supplementary Figure S1. p107 associates specifically with a subset of tRNA genes in IMR90 cells. (A) Heatmap depicting p107 binding 10kb either side of hg38 tRNA genes (left) or sno/miRNA genes (right) in IMR90 cells. (B) Average signal intensity of p107 binding at tRNA genes (pink) or sno/miRNA genes (purple) in IMR90 cells.

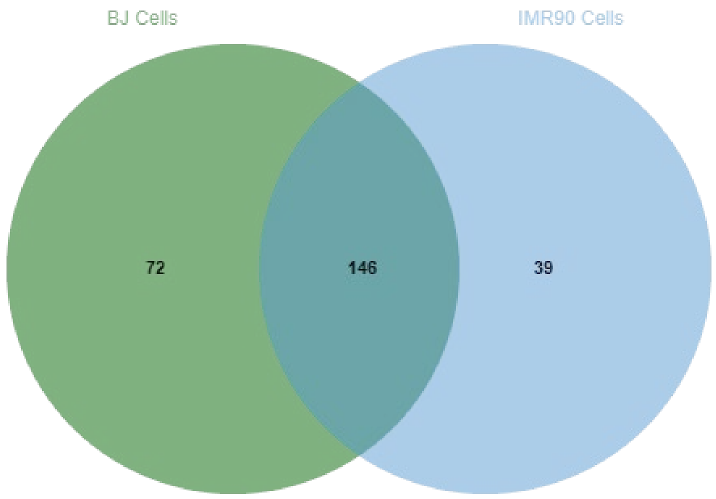

Supplementary Figure S2. Most tRNA genes that associate with RB above threshold are common between BJ and IMR90 lines of human diploid fibroblasts.

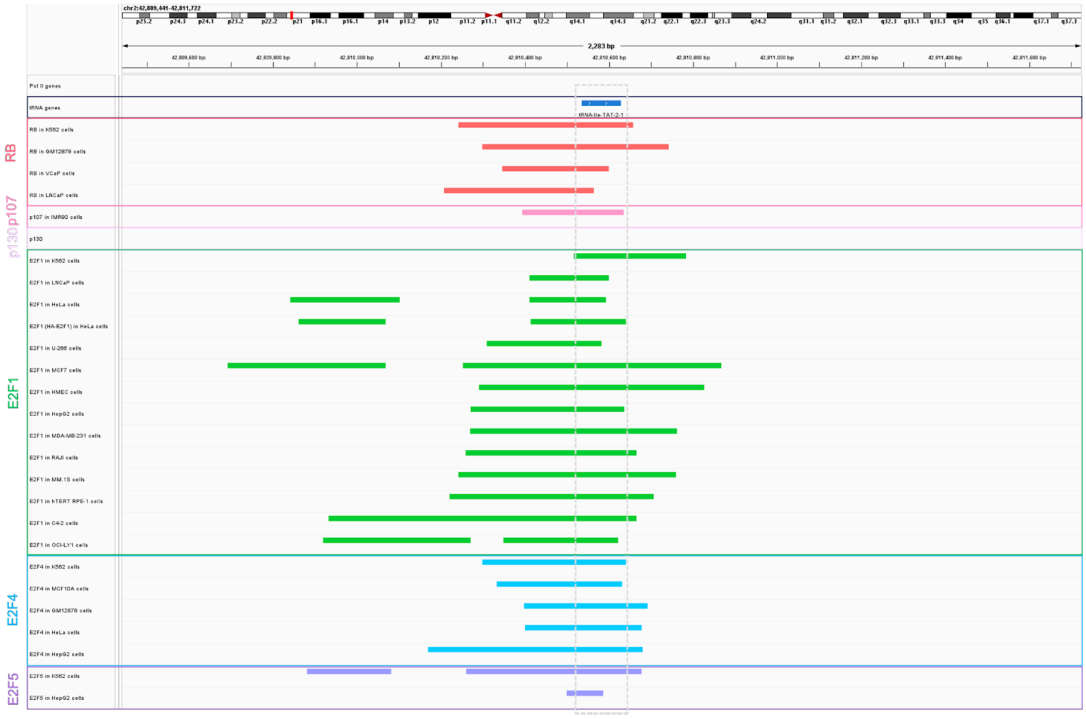

Supplementary Figure S3. ChIP-ATLAS peak-call data for RB and E2F factors at a tRNA gene. Data are shown in the IGV genome browser for settings of the “Peak Browser” web page (https://chip-atlas.org/peak_browser). Bars represent the peak regions at tRNA-Ile-TAT-2-1 (blue) and are shown for RB (red), p107 (pink), p130 (light pink), E2F1 (green), E2F4 (pale blue) and E2F5 (purple) with cell types indicated on the left hand side.

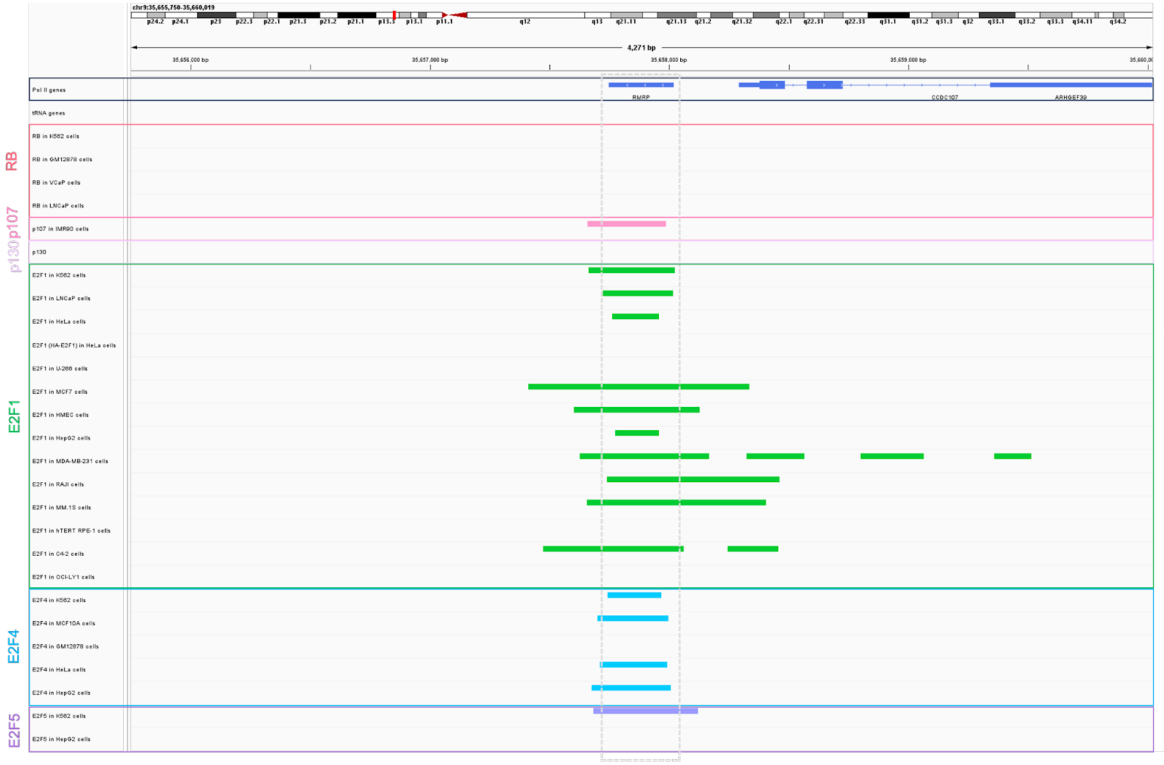

Supplementary Figure S4. ChIP-ATLAS peak-call data for RB and E2F factors at the RMRP gene. Data are shown in the IGV genome browser for settings of the "Peak Browser" web page (https://chip-atlas.org/peak_browser). Bars represent the peak regions at RMRP and are shown for RB (red), p107 (pink), p130 (light pink), E2F1 (green), E2F4 (blue) and E2F5 (purple), with cell types indicated on the left hand side.

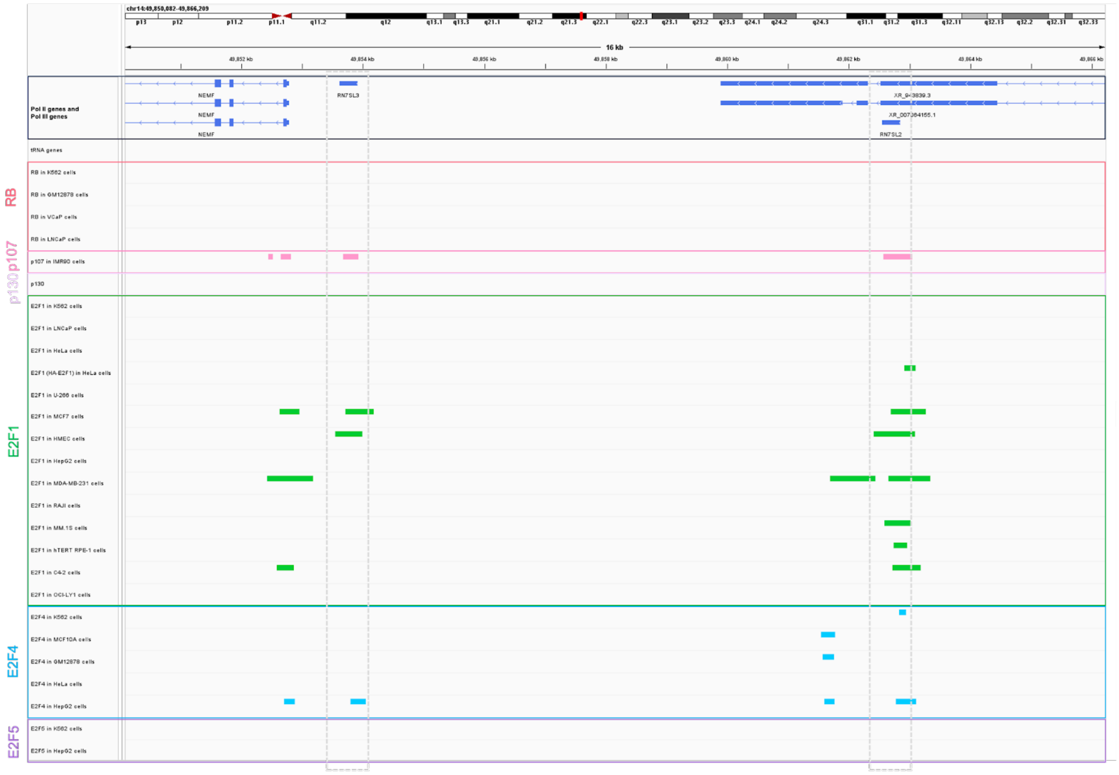

Supplementary Figure S5. ChIP-ATLAS peak-call data for RB and E2F factors at two 7SL genes. Data are shown in the IGV genome browser for settings of the "Peak Browser" web page (https://chip-atlas.org/peak_browser). Bars represent the peak regions at RN7SL3 (left) and RN7SL2 (right) and are shown for RB (red), p107 (pink), p130 (light pink), E2F1 (green), E2F4 (blue) and E2F5 (purple) with cell types indicated on the left hand side.
